# Contrasting leaf transcriptomic responses to drought and heat stress in the desert CAM species *Mesembryanthemum forsskalii*

**DOI:** 10.3389/fpls.2026.1805066

**Published:** 2026-03-17

**Authors:** Faten Dhawi, Sumayah I. Alsanie

**Affiliations:** 1 Agricultural Biotechnology Department, College of Agricultural and Food Sciences, King Faisal University, Al-Ahsa, Saudi Arabia; 2 Department of Biology, College of Science, Imam Abdulrahman Bin Faisal University (IAU), Dammam, Saudi Arabia; 3 Basic and Applied Scientific Research Center, College of Science, Imam Abdulrahman Bin Faisal University (IAU), Dammam, Saudi Arabia

**Keywords:** *Mesembryanthemum forsskalii*, facultative CAM, heat stress, drought stress, transcription factors, rhizosphere metagenome, desert plant adaptation, thermotolerance

## Abstract

**Introduction:**

Dryland ecosystems are increasingly exposed to extreme heat and prolonged water limitation. Facultative crassulacean acid metabolism (CAM) enables certain desert plants to enhance water-use efficiency and adjust carbon assimilation under stress conditions. *Mesembryanthemum forsskalii* Hochst. ex Boiss. (Aizoaceae; locally known as Samh) is a hyper-arid adapted species native to Saudi Arabia, yet genomic and transcriptomic resources for this plant remain scarce. This study aimed to generate foundational genomic resources and characterize transcriptional responses to drought and heat stress.

**Methods:**

We integrated rhizosphere metagenomics and leaf transcriptomics. A genome-resolved rhizosphere metagenome was generated from mature field-grown plants. In parallel, micropropagated plants were exposed under controlled conditions to progressive drought (17 days without irrigation) or acute heat shock (55 °C for 120 min), each compared with well-watered controls. RNA sequencing generated 123.77 Gb raw data and 121.96 Gb clean reads after quality filtering. Differential gene expression was identified using thresholds of |log_2_FC| ≥ 2 and FDR ≤ 0.05, followed by transcription factor profiling and KEGG pathway annotation.

**Results:**

Heat stress induced substantially broader transcriptional reprogramming than drought. A total of 1,348 genes were differentially expressed under heat stress, compared with 84 genes under drought. Heat exposure strongly increased the expression of transcription factor families including B3 (20.00-fold relative to drought), bHLH (22.65-fold), and bZIP (8.94-fold). KEGG pathway analysis revealed expanded representation of metabolic pathways under heat, including secondary metabolite biosynthesis, ribosome function, carbon metabolism, and endoplasmic reticulum protein processing. Rhizosphere binning recovered archaeal and bacterial genomes affiliated with stress-tolerant lineages, providing the first microbial genomic framework associated with *M. forsskalii*.

**Discussion:**

These results demonstrate a heat-dominant transcriptional response in *M. forsskalii* and provide the first integrated transcriptomic and rhizosphere metagenomic resources for this desert facultative CAM species. Heat-inducible transcription factors, particularly B3 and NAC families, emerge as promising targets for improving thermotolerance and water-use efficiency in crops.

## 1 Introduction

Rising temperatures and increasing evaporative demand are intensifying water limitation across drylands, where plants are frequently exposed to drought and heat within the same growing season. These stresses can act independently or interactively, affecting carbon gain, protein stability, membrane integrity, and oxidative balance. Understanding stress-tolerance strategies in desert-adapted taxa remains a priority for climate-resilient agriculture and ecosystem sustainability. Plants face escalating threats from drought and heat stress under climate change (Öztürk et al., 2019). Desert-adapted species employing Crassulacean Acid Metabolism (CAM) photosynthesis are exceptionally resilient because nocturnal CO_2_ fixation dramatically improves water-use efficiency while minimizing transpirational water loss during the hottest periods of the day (Amin et al., 2019).

The Aizoaceae include multiple CAM-capable succulents and have served as models for inducible CAM regulation (e.g., *Mesembryanthemum forsskalii). Mesembryanthemum forsskalii* Hochst. ex Boiss. (Aizoaceae), locally known as Samh, is a desert−adapted succulent distributed in parts of North Africa and the Arabian Peninsula, where it experiences intense heat, high irradiance, and seasonal water limitation. Recent phenotypic characterization of Saudi Arabian populations revealed a suite of pre-adaptive traits that enable survival in environments routinely exceeding 45 °C with annual precipitation below 100 mm: decumbent-to-erect terete succulent branches, specialized epidermal bladder cells for water and ion storage, opposite decussate phyllotaxis, dark violet leaf margins, a short life cycle terminating in rapid post-flowering dehydration by late May, and winged seeds optimized for wind dispersal (Alsanie, 2025). These structural adaptations, combined with high seed protein content and documented medicinal potential (Al Faris et al., 2011), make *M. forsskalii* a valuable yet underexploited genetic resource for arid-land agriculture and nutritional security. Although the congener *M. crystallinum* has served as a model for salinity- and drought-induced CAM engagement, revealing activation of core CAM genes such as *Ppc1* and osmoprotectant biosynthesis genes (*Imtl*) (Guan et al., 2020) and conserved drought-responsive transcription factor cascades (e.g., AP2/ERF, WRKY) (Agarwal et al., 2006; Baillo et al., 2019), these studies predominantly address single-stress regimes and largely omit heat or multi-stress interactions (Chinnusamy and Zhu, 2009). In contrast, transcriptomic analyses in C3/C4 species under combined drought and heat stress highlight synergistic activation of heat-shock factors, ROS-scavenging systems, hormonal crosstalk, and suppression of photosynthesis (Katoh and Standley, 2013; Dhawi, 2023; Dhawi, 2024). However, the unique diurnal separation of carboxylation and decarboxylation in CAM plants is expected to generate distinct regulatory networks, particularly under thermal stress that impairs nocturnal CO_2_ uptake and malate remobilization (Stieglmeier et al., 2014).

Despite its ecological relevance and emerging interest for micropropagation and biotechnological utilization (Blankenberg et al., 2010; Alsanie, 2025), the molecular basis of stress acclimation in *M. forsskalii* remains far less resolved than in established CAM models. In particular, it is unclear how heat versus drought cues differentially shape CAM-associated gene expression, regulatory transcription factor networks, and downstream protective pathways. Filling this gap will strengthen mechanistic understanding of facultative CAM performance under desert stress regimes and broaden the suite of CAM resources for applied research., offering novel targets for engineering thermotolerance in crops (Bolger et al., 2014; Ewels et al., 2016).

Plant-associated microbiomes can modulate nutrient cycling and plant stress tolerance in arid environments. Due to differing sample sources, our microbiome data provide baseline context rather than causal evidence for influencing host transcriptome responses to acute heat stress; matched sampling designs are needed for direct integration and causality testing. In this study, we integrate rhizosphere metagenomics and leaf transcriptomics to elucidate the molecular and microbial mechanisms underpinning *M. forsskalii* resilience to drought and heat stress. We aimed to:

1. Generate a genome-resolved baseline profile of the *M. forsskalii* rhizosphere microbiome from field-grown mature plants using shotgun metagenomics and MAG recovery.
2. Build a leaf transcriptome resource for *M. forsskalii* and quantify differential expression under progressive drought vs. acute heat shock in controlled conditions.
3. Identify major functional categories and TF-family signatures associated with the larger heat-driven transcriptomic shift.

These findings would establish *M. forsskalii* as a new model for multi-stress tolerance in facultative CAM plants and provide actionable molecular targets for developing climate-resilient crops suited to arid and semi-arid agroecosystems.

## 2 Materials and methods

### 2.1 Plant material and experimental design

*Mesembryanthemum forsskalii Hochst. ex* Boiss. (Aizoaceae; local name: Samh) plants used in this study originated from the same natural population in Dumat Al-Jandal, Al-Jouf Province, Saudi Arabia (29°41′22.0″N, 39°35′41.8″E) that was phenotypically characterized by Alsanie (2025). Species identity was confirmed using Migahid’s Flora of Saudi Arabia (1996) and voucher specimens are retained at Imam Abdulrahman Bin Faisal University Herbarium.Uniform two-month-old plantlets were generated via the optimized *in vitro* micropropagation protocol established by Alsanie (2025) using receptacle explants cultured on Murashige and Skoog (MS) medium supplemented with 2.0 mg L^-^¹ 6-benzyladenine (BA), which yielded the highest shoot induction rate and full morphological fidelity to wild-type plants (terete succulent stems, epidermal bladder cells, violet leaf margins) (Alsanie, 2025). Regenerants were rooted on half-strength MS medium, acclimatized for four weeks in a controlled greenhouse (27 ± 2 °C, 60 ± 5% RH, 16 h photoperiod, 250 µmol m^-^² s^-^¹ supplemental LED lighting), and then transferred to 15 cm pots containing a sterile peat moss:perlite mixture (1:1 v/v).After acclimatization, 45 uniformly sized plants were randomly allocated to three treatments (n = 15 plants per treatment; five biological replicates, each comprising three plants) in a randomized complete block design:

- Control (C): daily irrigation to maintain 80–100% field capacity.
- Heat stress (H): single acute exposure to 55 °C for 120 min in a growth chamber (40–50% RH, 300 µmol m^-^² s^-^¹ PAR), followed by recovery under control conditions.
- Drought stress (D): complete withholding of irrigation for 17 days until soil moisture fell below 10% of field capacity and moderate wilting was observed (Gurevich et al., 2013).

### 2.2 Sample collection and preservation

Immediately after termination of each stress treatment, the third and fourth fully expanded leaves (counted from the apex) were harvested, pooled, flash-frozen in liquid nitrogen, and transferred to RNA-later™ Stabilization Solution (Thermo Fisher Scientific). Samples were infiltrated overnight at 4 °C and stored at –80 °C until RNA extraction, following the preservation protocol validated for *M. forsskalii* by Alsanie (2025).

### 2.3 RNA extraction, library preparation, and sequencing

Total RNA was isolated from 100 mg frozen leaf tissue (three biological replicates per treatment) using the RNeasy Plant Mini Kit (QIAGEN) with on-column DNase I digestion, following Dhawi (2023). RNA integrity (RIN > 7.5) was verified on an Agilent 2100 Bioanalyzer. Poly(A) mRNA libraries were constructed using the TruSeq RNA Library Prep Kit v2 (Illumina) and sequenced on an Illumina NovaSeq 6000 platform (2 × 150 bp), generating 123.77 Gb raw data.

### 2.4 Transcriptome assembly, differential expression, and functional annotation

Raw reads were trimmed with Trim Galore v0.6.2, yielding 121.96 Gb clean data. *De novo* assembly was performed using Trinity v2.11.55 (k-mer = 25), and redundancy reduced with CD-HIT-EST (90% similarity). Clean reads were mapped using Bowtie2 implemented in RSEM, and transcript abundance quantified as TPM and RPKM. Differential expression analysis was conducted with DESeq2 v1.30.0 (Benjamini–Hochberg adjusted p ≤ 0.05, |log_2_ fold-change| ≥ 2). Functional annotation used BLASTx against NCBI NR (e-value ≤ 10^-5^), with GO and KEGG enrichment using topGO v2.30.1 and clusterProfiler v4.6.2 (FDR ≤ 0.05).

### 2.5 Functional annotation and KEGG pathway summaries

Functional annotation was performed using sequence similarity searching against protein databases with downstream Gene Ontology and KEGG assignment. KEGG pathway representation for stress-responsive gene sets was summarized for drought and heat conditions and used to support functional interpretation of treatment-associated transcriptional changes.

### 2.6 Transcription factor identification and phylogenetic analysis

Transcription factors were identified from UniProt annotations (Casimiro-Soriguer et al., 2017) and manually curated. Protein alignments were generated with MAFFT v7 (Katoh and Standley, 2013; Price et al., 2010), phylogenetic trees constructed with FastTree v2.1 (Blankenberg et al., 2010), and visualized in iTOL. Heatmaps of TF expression were produced using ComplexHeatmap and circlize packages in R.

### 2.7 Rhizosphere metagenomics: sampling, assembly, binning, and annotation

Rhizosphere soil (0.5–1 cm from roots) was collected in April 2023 from three mature field-grown *M. forsskalii*plants at the original Dumat Al-Jandal site. DNA extraction, library preparation, and Illumina NovaSeq 6000 PE150 sequencing (~10 Gb raw data per sample) were performed as previously described (Yang et al., 2017). Quality filtering used FastQC/MultiQC (Blankenberg et al., 2010; Ewels et al., 2016; UniProt Consortium, 2007), trimming used Trimmomatic v0.39 (Bushnell, 2014), and *de novo* assembly used MEGAHIT v1.1.3 (Kang et al., 2019). Taxonomic binning and refinement were carried out using MetaWRAP v1.3.2 integrating Kraken2, MetaBAT2, MaxBin2, and CONCOCT (Alneberg et al., 2014; Parks et al., 2015). Only high- and medium-quality MAGs assessed by CheckM v1.0.12 (Parks et al., 2015) were retained. Functional annotation employed Prokka v1.12, Swiss-Prot (Casimiro-Soriguer et al., 2017), and Sma3s v2 (Hernández-de-Diego et al., 2018), with pathway reconstruction in iPath3.0 (Truong et al., 2015).

### 2.8 Data availability

Leaf RNA-seq data are available from the NCBI Sequence Read Archive under accession PRJNA1073520.

## 3 Results

### 3.1 Taxonomic composition of the rhizosphere microbiome

**Figure 1:**
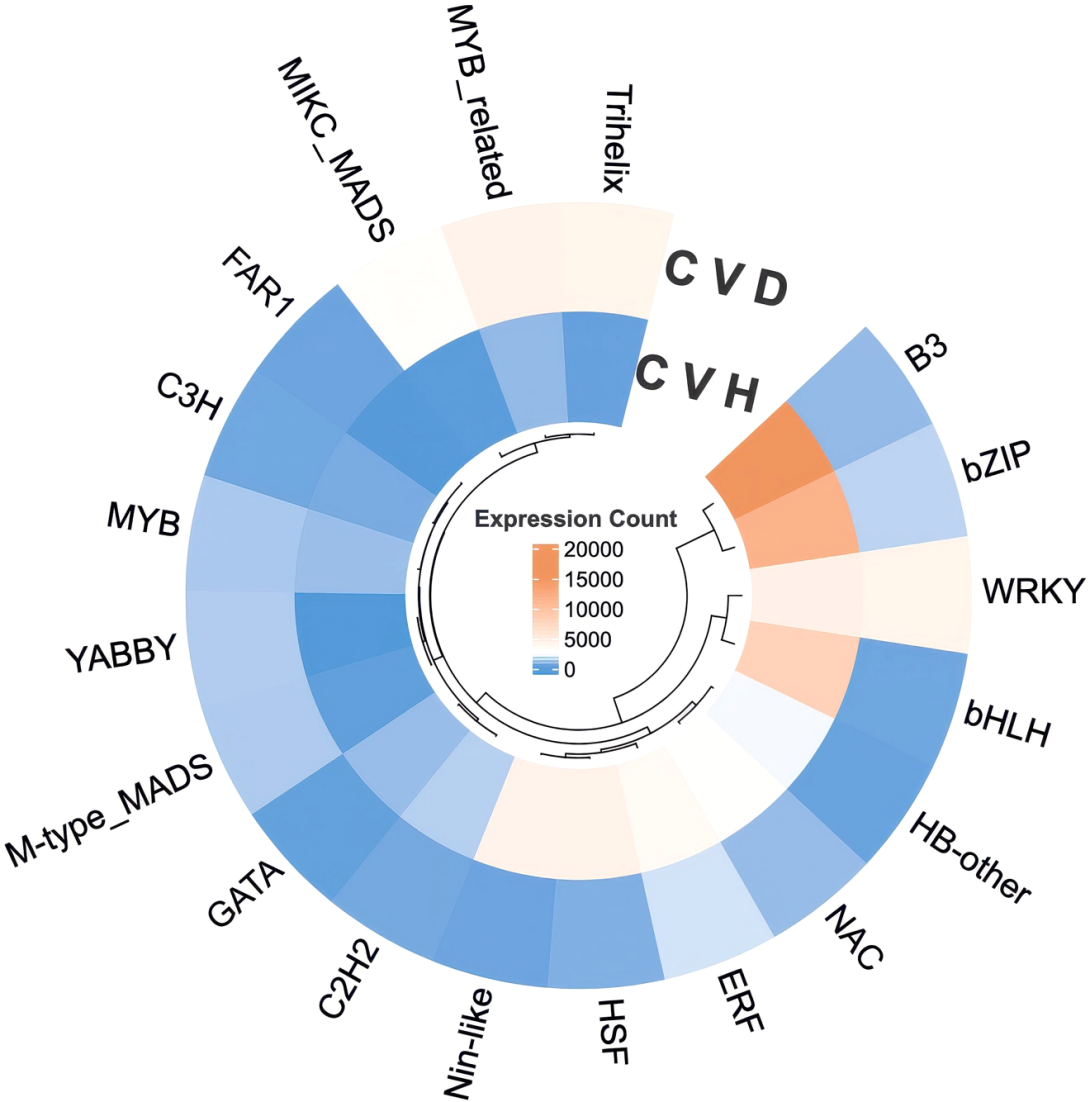
Blobplot depicting contig bin membership from metagenomic analysis of M. forskahlii rhizosphere samples. This scatter plot displays the distribution of contigs based on their GC content (x-axis, ranging from 0.2 to 0.8) and contig abundance (y-axis, log-scaled from 1 to 10,000), highlighting bin assignments derived from metagenomic analysis of M. forskahlii rhizosphere samples.

Shotgun metagenomic sequencing of rhizosphere soil collected from three mature field plants yielded an assembled contig set that was partitioned into multiple genome bins together with an unbinned fraction (**Figure 1**). The blobplot summarizes contig-level GC content and abundance (log scale) and shows discrete clusters corresponding to individual bins, consistent with genome-resolved recovery of taxonomically distinct community members. MegaBLAST-based assignments indicated that two bins (bin.5.permissive.fa and bin.6.strict.fa) were affiliated with ammonia-oxidizing archaea in the family Nitrososphaeraceae (Nitrososphaerota), which formed low-to-moderate GC clusters at comparatively low abundances. A distinct, higher-GC and higher-abundance cluster corresponded to bin.3.orig.fa, assigned to Xanthomonadaceae (Gammaproteobacteria), whereas bin.9.orig.fa was assigned to Rubrobacteraceae (Actinomycetota; class Rubrobacteria) and formed a separate mid-GC cluster. An additional bin was assigned more broadly to Actinomycetota (bin.2.orig.fa), while several bins remained classified only at the domain level (Bacteria; bin.1.orig.fa, bin.4.strict.fa, and bin.8.orig.fa) (**Table 1**).

**Table 1:** Bin taxonomy from **Figure 1** (the resulting contigs of metagenomic analysis of rhizosphere samples from Mesembryanthemum forskahlii) assigned using MegaBLAST.

Assembly	Taxonomic assignment
bin.6.strict.fa	Archaea;Nitrososphaerota;Nitrososphaeria;Nitrososphaerales;Nitrososphaeraceae
bin.5.permissive.fa	Archaea;Nitrososphaerota;Nitrososphaeria;Nitrososphaerales;Nitrososphaeraceae
bin.9.orig.fa	Bacteria;Actinomycetota;Rubrobacteria;Rubrobacterales;Rubrobacteraceae
bin.3.orig.fa	Bacteria;Pseudomonadota;Gammaproteobacteria;Xanthomonadales;Xanthomonadaceae
bin.4.strict.fa	Bacteria
bin.8.orig.fa	Bacteria
bin.2.orig.fa	Bacteria; Actinomycetota
bin.1.orig.fa	Bacteria

### 3.2 Transcription factor expression and regulation

Transcription factor (TF) family summaries highlighted large contrasts in normalized expression between drought and heat treatments (**Table 2**).

**Table 2:** Normalized expression counts for each transcription factor family compared to the control across each stressed group (D, Drought, H, Heat) in Mesembryanthemum forskahlii leaves.

Transcription factor family	Normalized expression counts in leaves in D: drought group	Normalized expression counts in leaves in H: heat	Fold change (H/D)
B3	750	15002	20.00
bZIP	1298	11599	8.94
bHLH	350	7926	22.65
WRKY	4046	4327	1.07
HSF	530	4147	7.82
Nin-like	315	4093	12.99
ERF	1581	3410	2.16
NAC	807	2677	3.32
HB-other	287	2043	7.12
C2H2	391	1200	3.07
MYB	1046	841	0.80
GATA	216	829	3.84
MYB_related	4082	748	0.18
C3H	354	439	1.24
Trihelix	4034	260	0.06
M-type_MADS	1084	183	0.17
MIKC_MADS	2828	128	0.05
FAR1	318	91	0.29
YABBY	1126	69	0.06

Quantitatively, the strongest heat-associated increases (H/D) were observed for bHLH (7,926 vs 350; 22.65-fold), B3 (15,002 vs 750; 20.00-fold), Nin-like (4,093 vs 315; 12.99-fold), bZIP (11,599 vs 1,298; 8.94-fold), and HSF (4,147 vs 530; 7.82-fold). Additional families increased under heat with more moderate ratios, including NAC (3.32-fold), C2H2 (3.07-fold), GATA (3.84-fold), ERF (2.16-fold), and HB-other (7.12-fold) (**Table 2**).

In contrast, several TF families exhibited lower normalized counts under heat than under drought (H/D < 1), including Trihelix (0.06), YABBY (0.06), MIKC_MADS (0.05), M-type_MADS (0.17), MYB_related (0.18), FAR1 (0.29), and MYB (0.80), suggesting stress-associated suppression of some growth and developmental regulators during acute heat exposure (**Table 2**).

**Figure 2:**
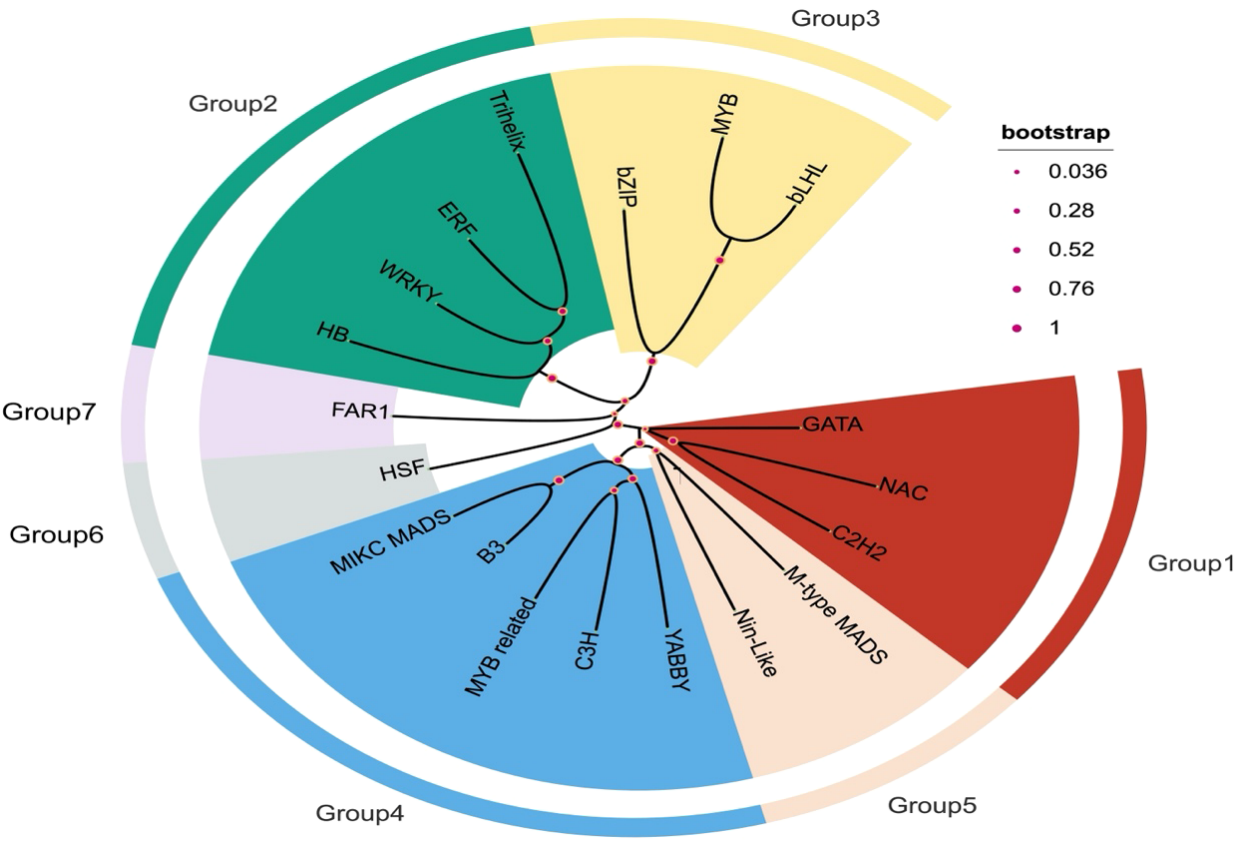
This circular phylogenetic tree illustrates the clustering of transcription factor (TF) families in M. forskahlii based on sequence similarity and expression profiles under drought and heat stress. The tree is segmented into seven groups (Group1 to Group7), each representing a distinct evolutionary clade. TF families are marked with lines extending from the center, reflecting their evolutionary relationships. Bootstrap values (0.036, 0.28, 0.52, 0.76, 1), displayed as colored dots at key nodes, indicate the statistical confidence of each cluster, with a value of 1 signifying the highest reliability.

A circular TF clustering visualization organized families into seven major groups (**Figure 2**). Group 3 contained B3, bZIP and bHLH, matching the most heat-enriched families in **Table 2**, whereas Group 6 was dominated by HSF. Other groups contained mixtures of stress-responsive (e.g., ERF, WRKY, NAC) and development-associated (e.g., MADS, YABBY) families (**Figure 2**).

**Figure 3:**
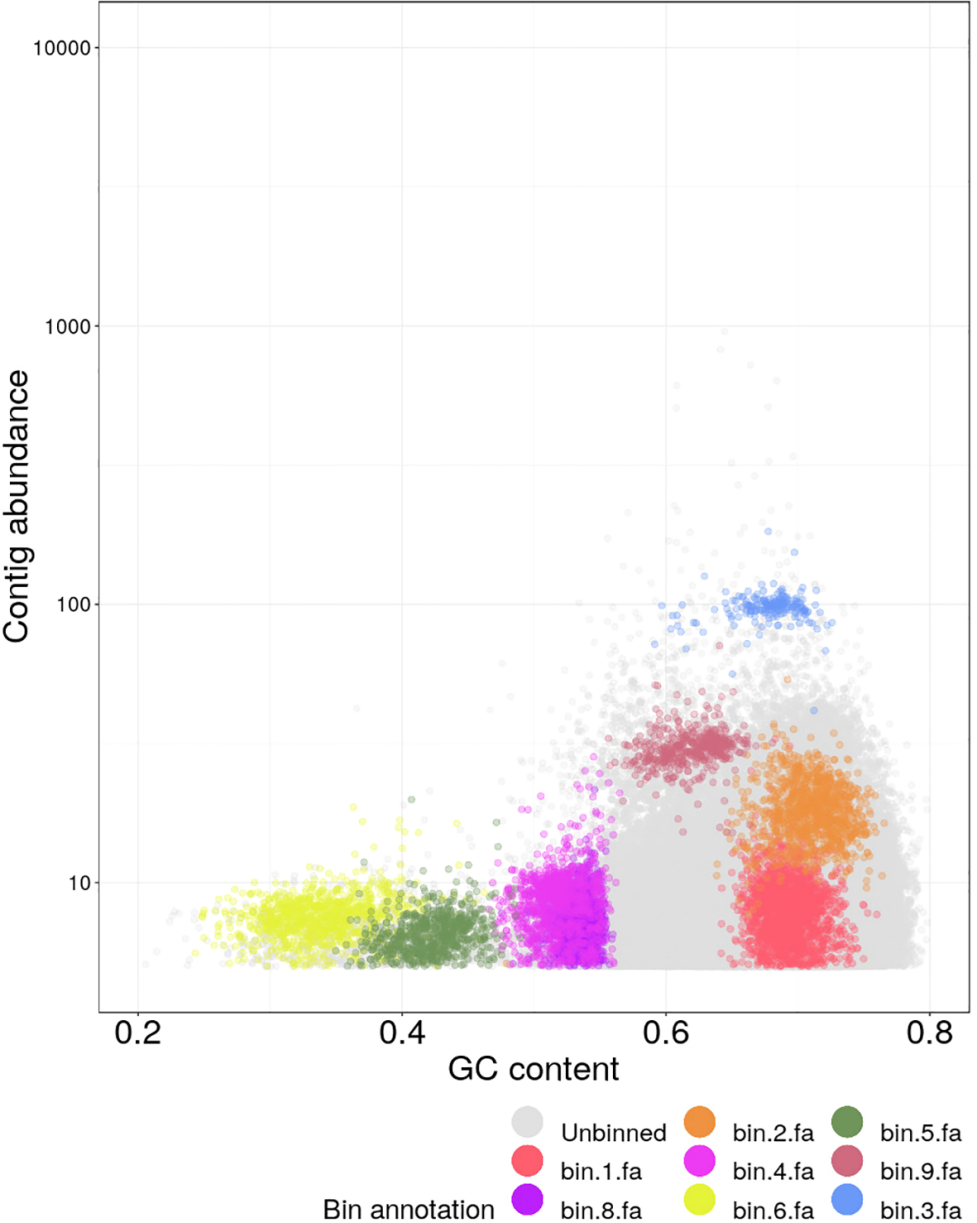
Circular heatmap of normalized expression counts for transcription factor Ffamilies relative to control across stressed groups (C vs. D, Drought and C vs. H, Heat) in Mesembryanthemum forskahlii. Each segment represents a TF family, with the radial length and color intensity indicating expression levels (ranging from 0 to 20,000 counts, with a color gradient from blue [low] to orange [high]). The inner ring (CVD) reflects expression changes under drought stress, while the outer ring (CVH) indicates changes under heat stress. Notable high-expression families under heat include B3 (15,002 counts), bZIP (11,599 counts), and bHLH (7,926 counts).

Consistent with the quantitative summary, the circular heatmap representation (**Figure 3**) shows higher relative signal in the heat ring (C vs H) than the drought ring (C vs D) for the most heat-enriched TF families (e.g., B3, bHLH, bZIP, HSF), while several families with H/D < 1 exhibit stronger drought-associated signal (**Figure 3**; **Table 2**).

### 3.3 Differential gene expression under heat and drought stress

**Figure 4:**
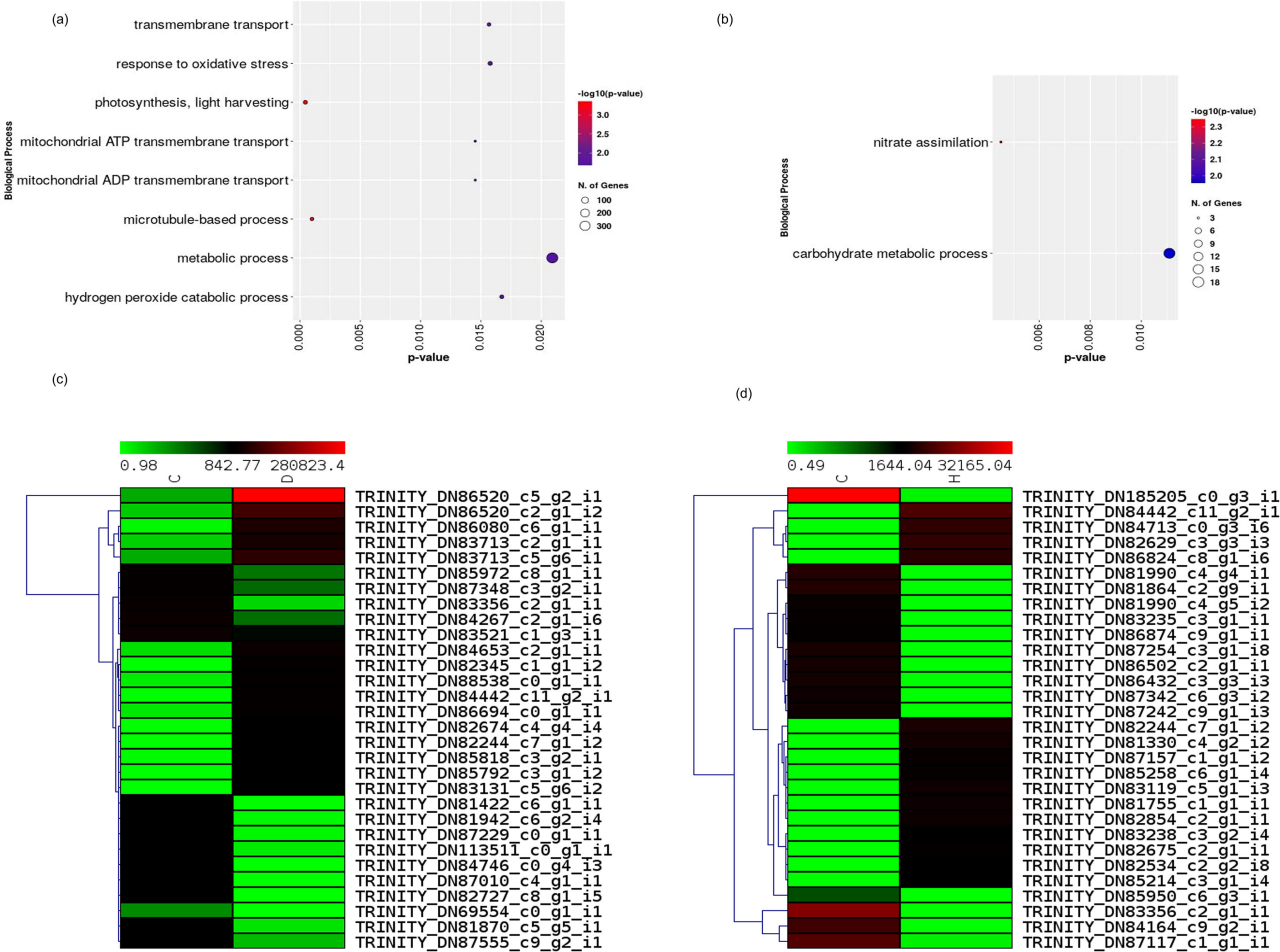
Biological process enrichment and gene expression heatmaps highlighting stress-specific responses in leaves of Mesembryanthemum forsskalii under progressive drought and acute heat shock. **(A)** Dot plot of enriched Gene Ontology (GO) biological processes under progressive drought stress (compared to control conditions). Enrichment significance is shown as -log_10_(p-value) on the x-axis, with dot color intensity representing -log_10_(p-value) (redder for stronger significance) and dot size proportional to the number of genes in each term (legend: small circle = 3 genes, larger up to 300+). Key drought-enriched processes include nitrate assimilation, carbohydrate metabolic process, photosynthesis/light harvesting, and metabolic process. **(B)** Dot plot of enriched GO biological processes under acute heat shock stress (compared to control conditions). Axes, color, and size scales are analogous to panel **(A)**, with p-value range adjusted to the heat data. Prominent heat-enriched processes include transmembrane transport, response to oxidative stress, hydrogen peroxide catabolic process, mitochondrial ATP/ADP transmembrane transport, and related terms reflecting proteostasis, redox balance, and energy demands. **(C)** Hierarchical clustered heatmap of normalized expression (log-transformed or similar scaled counts) for selected differentially expressed unigenes (Trinity assemblies) under drought stress (control vs. drought comparison). Rows represent individual unigenes (labeled with Trinity IDs); columns represent samples/conditions (likely grouped as control left, drought right, though exact grouping inferred from clustering). Color scale ranges from green (low relative expression) through black (intermediate) to red (high relative expression), with dendrograms indicating hierarchical clustering of rows (genes) and columns (samples). Compact clustering on the left suggests a limited, coherent drought-responsive signature. **(D)** Hierarchical clustered heatmap of normalized expression for selected differentially expressed unigenes under acute heat shock (control vs. heat comparison). Layout, color scale, and clustering analogous to panel **(C)**. Extensive red-black-green patterns and broader separation in clustering reflect the larger magnitude and breadth of heat-induced transcriptional reprogramming, consistent with 1,348 DEGs versus only 84 under drought.

Leaf RNA-seq generated 123.77 Gb raw data and 121.96 Gb clean data after trimming. Differential expression analysis revealed a pronounced contrast between stress regimes: heat stress was associated with 1,348 differentially expressed genes (DEGs), whereas drought was associated with 84 DEGs under the applied thresholds (see Methods). This disparity indicates that acute heat shock elicited substantially broader transcriptomic remodeling than the progressive drought regime at the endpoint sampling time used here. The functional breadth of the heat response is reflected in downstream functional category mapping and pathway assignment patterns (**Figure 4**; **Table 3**).

**Table 3:** The number of genes associated with KEGG pathways in Mesembryanthemum forskahlii leaves under drought and heat conditions.

Kegg_pathways	Number_of_Unigenes in heat stress	Number_of_Unigenes in drought stress
Metabolic pathways	1348	84
Biosynthesis of secondary metabolites	681	47
Ribosome	351	40
Carbon metabolism	248	18
Protein processing in endoplasmic reticulum	179	5
Biosynthesis of amino acids	165	11
Biosynthesis of cofactors	148	7
Spliceosome	145	3
Endocytosis	138	2
Oxidative phosphorylation	137	5
Thermogenesis	134	5

### 3.4 Functional category patterns and expression clustering under drought vs heat

**Figure 4** summarizes biological-process associations and representative gene-expression patterns under drought and heat. Under drought, the biological-process dot plot (**Figure 4A**) indicates that the comparatively small drought-responsive gene set is primarily associated with nitrate assimilation and carbohydrate metabolic process, with relatively few transcripts contributing to each term compared with the heat response. In contrast, the heat biological-process plot (**Figure 4B**) shows a broader and more complex functional footprint, with terms spanning metabolic process, transmembrane transport, response to oxidative stress, hydrogen peroxide catabolic process, and mitochondrial ATP/ADP transmembrane transport, consistent with extensive heat-associated remodeling of cellular homeostasis.

The expression heatmaps in **Figures 4C, D** further emphasize the contrast between stresses. The Control vs Drought heatmap (**Figure 4C**) shows clear clustering of transcripts into groups with opposing expression patterns between control and drought, but the overall signature is comparatively compact, consistent with the smaller number of drought DEGs. The Control vs Heat heatmap (**Figure 4D**) displays a more pronounced separation between conditions across a larger set of transcripts, reflecting the much stronger transcriptional reprogramming induced by heat.

### 3.5 KEGG pathway representation of stress-responsive genes

To contextualize stress-responsive transcripts within major pathways, DEGs were mapped to KEGG functional classes (**Table 3**). Across all listed categories, the number of mapped unigenes is substantially higher under heat than drought, including Metabolic pathways (1348 vs 84), Biosynthesis of secondary metabolites (681 vs 47), Ribosome (351 vs 40), and Carbon metabolism (248 vs 18). Several pathway classes show particularly large heat-to-drought contrasts, including Spliceosome (145 vs 3), Endocytosis (138 vs 2), and Protein processing in endoplasmic reticulum (179 vs 5), indicating broader representation of transcripts linked to RNA processing (Paliwal et al., 2021), trafficking, and proteostasis-related functions under heat relative to drought under the applied conditions (Williams et al., 2010). **Table 3** reflects pathway assignment/representation rather than a statistical pathway enrichment test (Gao et al., 2021; Gross et al., 2013; Suzuki, 2015).

## 4 Discussion

### 4.1 Baseline rhizosphere genome bins provide microbial genomic context but do not support treatment-resolved microbiome claims

The rhizosphere metagenome analysis provides baseline genomic context for microbes associated with mature field-grown *M. forsskalii*. The recovery of archaeal bins affiliated with Nitrososphaeraceae (ammonia-oxidizing archaea) and bacterial bins assigned to Rubrobacteraceae and Xanthomonadaceae (**Table 1**), together with distinct bin clusters visible in the blobplot (**Figure 1**), is consistent with a rhizosphere community containing stress-tolerant lineages. Desert and arid-plant rhizospheres commonly harbor stress-adapted microbial taxa and can contribute to host performance through nutrient cycling and stress buffering functions (Chinnusamy and Zhu, 2009; Boxall et al., 2020; Aloo et al., 2022; Alsanie, 2025; Zhang et al., 2024).

However, because rhizosphere samples were collected from mature field-grown plants (providing baseline context for the plant-associated microbial community) rather than from the controlled drought and heat treatments used for transcriptomics, the present dataset does not support claims of drought- vs. heat-specific microbiome separation, treatment-resolved functional enrichment, or direct causal contributions of identified stress-tolerant lineages (e.g., Nitrososphaeraceae) to host stress responses. Plant-associated microbiomes can influence nutrient cycling and enhance stress tolerance in plants growing in arid soils. However, directly linking microbiome composition (or specific lineages like ammonia-oxidizing archaea in Nitrososphaeraceae) to host transcriptome responses under acute stress requires matched sampling designs (i.e., profiling both microbiome and host transcriptome from the same plants, tissues, and time points). When different sample sources are used, microbiome data should be interpreted as providing baseline contextual information rather than evidence of causal drivers. Treatment-resolved sampling and quantitative abundance matrices would be required for ordination (e.g., PCA) and comparative microbiome inference.

### 4.2 Heat elicits a dominant transcriptional response relative to progressive drought under the tested regime

A central outcome of this study is the pronounced contrast in the magnitude and breadth of transcriptomic change between stress treatments. Acute heat shock was associated with 1,348 DEGs, whereas progressive drought at the endpoint sampling time was associated with 84 DEGs. This pattern is also reflected in the broader functional footprint observed under heat in **Figure 4** and the larger number of mapped pathway-associated transcripts in **Table 3**. A parsimonious interpretation is that the applied heat treatment triggers rapid, system-wide demands on protein stability, redox balance, membrane transport, and energy metabolism, thereby engaging a wide set of stress-response programs, whereas the drought regime and sampling timepoint captured a comparatively limited leaf transcriptomic signature (Xu et al., 2013; Alshareef et al., 2022; Ding et al., 2023). In the broader climate context, the increasing frequency and intensity of heat extremes in many regions underscores the relevance of such heat-dominant responses for arid and semi-arid systems (Dhawi et al., 2017; Wang et al., 2023).

Importantly, a smaller drought DEG set should not be interpreted as absence of physiological adjustment. In facultative CAM and succulent desert plants, drought tolerance may be supported by structural buffering (succulence and water storage), strong basal stress preparedness, tissue-specific regulation (e.g., roots), or temporal dynamics in which transcriptional peaks occur earlier than the endpoint sampled here (Blankenberg et al., 2010; Huang et al., 2022; Gross et al., 2013). Moreover, drought-responsive regulation is often governed by complex transcriptional networks and time-dependent signaling modules that may not be fully captured by a single endpoint sampling strategy (Guo et al., 2016; Dhawi, 2020). Thus, the present results are most appropriately framed as: under the tested drought duration and endpoint sampling, leaf transcriptional reprogramming was modest relative to the heat response.

### 4.3 Stress-regulatory transcription factor signatures support strong heat-associated reprogramming

**Figure 5:**
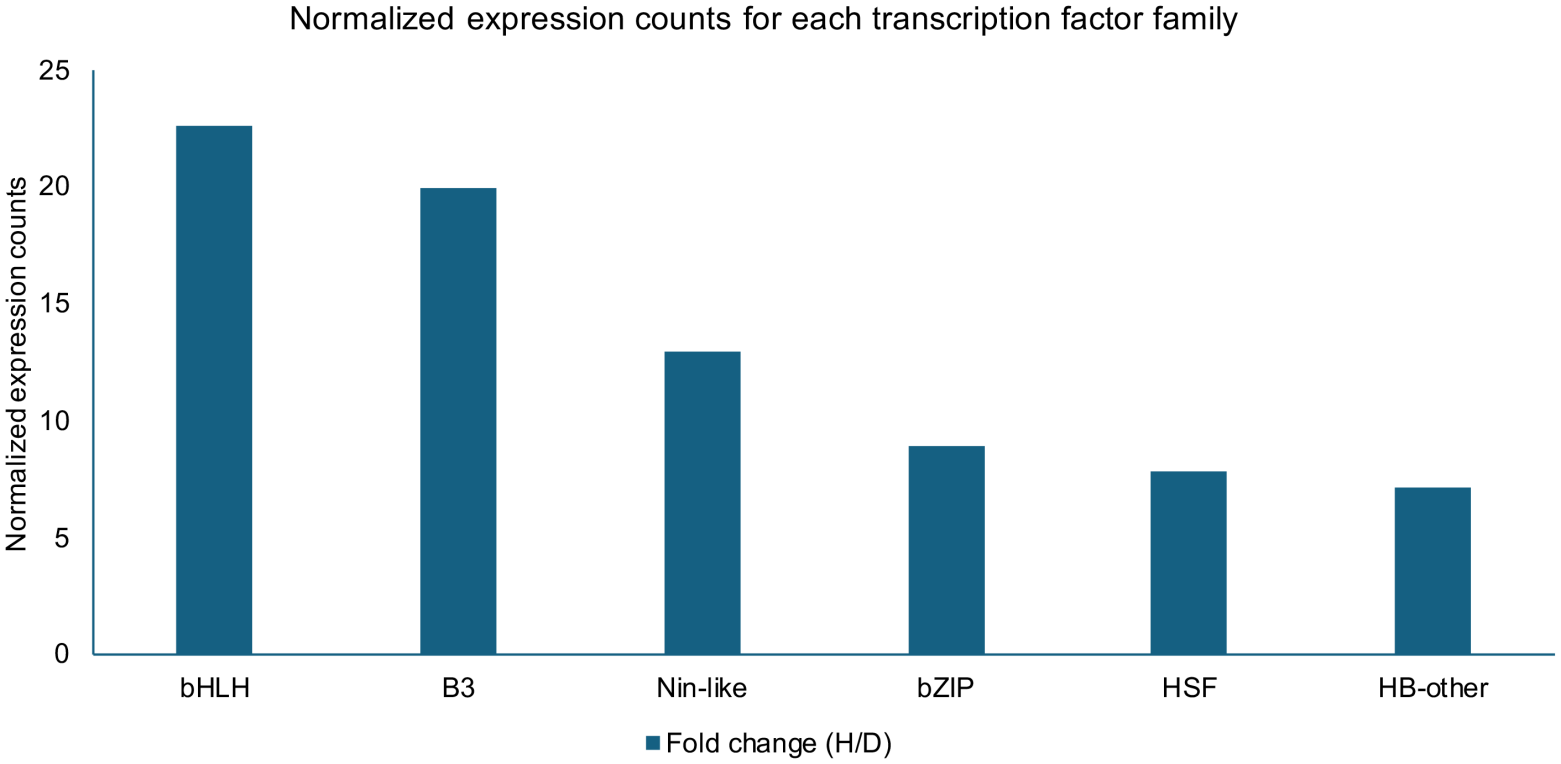
Heat/drought (H/D) fold-change in expression for upregulated transcription factor families under acute heat shock. Normalized fold-change (H/D) for TF families (bHLH, B3, Nin-like, bZIP, HSF, HB-other) exhibiting the strongest relative increases under heat, ranging from ~7 to 22-fold. Bars are ordered by descending H/D ratio (see **Table 2** for underlying normalized counts).

The TF-family summaries (**Table 2**; **Figure 5**) provide a coherent regulatory context for the heat-dominant response. Several families show large heat-associated increases in normalized expression counts, notably bHLH, B3, bZIP, Nin-like, HSF, and HB-other, along with moderate increases for NAC, ERF, C2H2, and GATA. These families are widely implicated in abiotic stress signaling, transcriptional control of protective metabolism, and maintenance of cellular homeostasis during high-temperature stress, including canonical heat-shock regulatory cascades (Alshareef et al., 2022). In contrast, drought-associated TF expression was comparatively limited, consistent with the smaller drought DEG set, and drought-responsive transcriptional programs frequently show tissue specificity and temporal regulation, particularly via ABA-dependent and ABA-independent networks (Guo et al., 2016; Mekureyaw et al., 2022).

Notably, **Table 2** also indicates that some TF families show limited change or lower heat-associated expression relative to drought (e.g., MYB_related, Trihelix, MADS-box groups).

Interestingly, several TF families, including Trihelix, YABBY, and certain MADS-box groups, exhibited relatively lower normalized expression under acute heat compared to drought (H/D < 1; **Table 2**). These families are frequently associated with developmental processes such as leaf morphogenesis (YABBY), organ polarity and growth regulation (Trihelix), and reproductive/vegetative development (MADS-box). Their relative suppression during heat shock may reflect a strategic resource allocation trade-off, whereby growth- and development-related transcriptional programs are deprioritized to favor rapid activation of survival-oriented responses, including heat-shock protein induction, proteostasis, and oxidative stress mitigation (Kaplan-Levy et al., 2012; Huot et al., 2014). This pattern aligns with established models of heat stress acclimation, in which plants transiently repress growth-promoting pathways to enhance short-term thermotolerance, particularly under acute exposures that impose immediate proteotoxic and redox challenges (Kaplan-Levy et al., 2012; Huot et al., 2014). In contrast, the more modest drought response (with limited DEG/TF changes) may permit greater retention of developmental regulation, consistent with the structural/temporal adaptations typical of drought-tolerant succulents like M. forsskalii.

This stress-specific TF pattern suggests that the heat response in *M. forsskalii* preferentially recruits specific regulatory modules rather than broadly elevating all TF classes, consistent with modular architecture of plant stress-regulatory networks (Guo et al., 2016; Mekureyaw et al., 2022). **Figures 2**, **3** complement **Table 2** by providing phylogenetic context and visualization of TF expression patterns, supporting the conclusion that multiple TF families contribute to heat-associated transcriptional restructuring.

### 4.4 Functional category mapping highlights oxidative stress, transport, and proteostasis-related processes under heat

The biological-process mapping and expression heatmaps (**Figure 4**) provide two complementary lines of evidence for stress-specific functional organization. First, the biological-process dot plots indicate that drought-responsive transcripts are concentrated in a small number of processes, particularly nitrate assimilation and carbohydrate metabolic process, whereas the heat response spans a wider range of cellular programs including transmembrane transport, response to oxidative stress, hydrogen peroxide catabolic process, and mitochondrial ATP/ADP transport. Second, the heatmaps show a compact control–drought signature (**Figure 4C**) versus a more extensive, condition-separating control–heat signature (**Figure 4D**), consistent with the DEG contrast. Collectively, these patterns align with established models in which acute heat stress imposes strong oxidative and proteostasis burdens that require broad cellular adjustment (Alshareef et al., 2022; Ding et al., 2023).

**Table 3** further supports this interpretation at the pathway-class level by showing substantially broader representation of heat-associated transcripts across KEGG categories, including metabolic pathways, secondary metabolite biosynthesis, ribosome, and carbon metabolism. Particularly large heat-to-drought differences for protein processing in endoplasmic reticulum, spliceosome, and endocytosis suggest that acute heat engages extensive transcript representation related to protein quality control, RNA processing, and cellular trafficking—functions commonly required for rapid acclimation when proteome integrity and membrane systems are challenged (Alshareef et al., 2022). Because **Table 3** reports pathway assignments rather than enrichment statistics, these results are best interpreted as an index of the breadth of pathway representation among stress-responsive transcripts.

Given that *M. forsskalii* is a facultative CAM species capable of shifting from C3 to CAM under certain abiotic stresses (e.g., drought or salinity, analogous to the well-studied congener M. crystallinum), we examined whether the extensive heat-responsive transcriptome (1,348 DEGs) and enriched representation of ‘metabolic pathways’ and ‘carbon metabolism’ in KEGG summaries included core CAM enzymes or indicated a reversion to C3-like patterns. Core CAM pathway components, such as phosphoenolpyruvate carboxylase (Ppc1/Ppc) for nocturnal CO_2_ fixation, were not prominently upregulated in the heat DEG set (or showed limited/no differential expression relative to controls). This suggests that acute heat shock does not induce a CAM-like transcriptional program in leaves under the tested regime. Instead, the broad metabolic reprogramming under heat encompassing carbon metabolism, secondary metabolites, and energy-related pathways aligns more closely with maintenance or enhancement of C3-like daytime photosynthetic and respiratory activity to support rapid acclimation and survival. Acute heat imposes immediate proteotoxic and oxidative demands that may favor generalized metabolic flexibility and energy allocation toward heat-shock protection (e.g., HSPs, redox balance) rather than the temporal and energetic commitment required for CAM cycling. This contrasts with drought, where structural adaptations (succulence) and potential delayed/tissue-specific CAM induction may explain the modest leaf transcriptional response observed here. Future targeted qPCR or proteomics on CAM marker genes (e.g., Ppc1, PPCK, malic enzyme isoforms) across diurnal cycles and stress combinations would clarify whether heat modulates CAM expression or promotes reversion to C3-dominant patterns as a survival mechanism in this desert facultative CAM plant.

### 4.5 Study limitations and implications for future work

Several limitations should be considered when interpreting these results. First, the drought response is based on an endpoint sampling strategy; temporal profiling and inclusion of root tissues may reveal additional drought-responsive programs not captured here, particularly for pathways that are strongly time dependent or tissue specific (Guo et al., 2016; Mekureyaw et al., 2022). Second, the study is primarily bioinformatics-based and does not include independent experimental validation (e.g., RT-qPCR), which would strengthen confidence in specific candidate regulators. Finally, while the rhizosphere metagenome provides valuable baseline genomic context for stress-tolerant lineages such as Nitrososphaeraceae (ammonia-oxidizing archaea commonly enriched in arid rhizospheres and linked to nitrogen cycling), the different sample sources preclude conclusions about their direct role in modulating host responses to acute heat shock (as observed in the leaf transcriptome) or progressive drought. A matched sampling design where rhizosphere microbiome and host transcriptome are profiled from the same experimental plants, tissues (e.g., roots/leaves), and time points under controlled stress treatments will be essential for rigorous microbiome–host integration and causality testing (Chinnusamy and Zhu, 2009; Aloo et al., 2022; Alsanie, 2025).

Such designs could specifically test whether these lineages directly assist the plant during acute heat shocks by, for example: (i) time-series sampling to capture dynamic microbial shifts alongside host gene expression (e.g., heat-shock proteins, oxidative stress pathways); (ii) microbiome manipulation experiments (e.g., inoculation with enriched Nitrososphaeraceae communities in gnotobiotic systems followed by acute heat exposure and assessment of plant outcomes like photosynthetic efficiency or ROS mitigation); or (iii) integrated multi-omics (metagenomics + metatranscriptomics of the microbiome with host RNA-seq) to link microbial functional gene expression to plant protection mechanisms. These approaches would help determine if Nitrososphaeraceae or similar taxa actively contribute to heat resilience via enhanced nitrogen availability, stress buffering, or other interactions.

Despite these constraints, the present work provides foundational transcriptome and genome-resolved rhizosphere resources for an under-studied desert facultative CAM plant and establishes a clear, data-supported conclusion: under the applied conditions, acute heat shock drives substantially broader transcriptional reprogramming in leaves than progressive drought, accompanied by strong heat-associated representation of multiple stress-regulatory TF families and broad pathway-class representation in functional mapping outputs (Dhawi et al., 2017; Alshareef et al., 2022; Huang et al., 2022).

## 5 Conclusions

This study provides foundational multi-omics resources for the desert facultative CAM plant *Mesembryanthemum forsskalii*, combining (i) a genome-resolved baseline rhizosphere metagenome from mature field plants and (ii) controlled-environment leaf transcriptomes generated under drought and acute heat stress. Across the applied conditions and endpoint sampling, acute heat shock elicited a markedly stronger transcriptomic response than progressive drought, evidenced by a much larger set of differentially expressed genes and broader functional category representation. Transcription factor profiling further supported a heat-dominant regulatory signature, with multiple TF families showing substantially higher heat-associated representation relative to drought. Functional category mapping and pathway assignment indicated that heat-responsive transcripts span diverse processes linked to metabolism, transport, oxidative stress responses, and protein homeostasis, including prominent representation of proteostasis-related pathways. The rhizosphere metagenome yielded distinct genome bins affiliated with stress-tolerant microbial lineages, including Nitrososphaeraceae, Rubrobacteraceae, and Xanthomonadaceae, providing baseline microbial genomic context for *M. forsskalii* in arid field conditions.

*Mesembryanthemum forsskalii* appears to cope with arid‐zone stress via a two-tier tolerance strategy. Tier 1 (drought) is dominated by low-cost, largely constitutive mechanisms—succulent tissues and ion/water storage structures, a rapid annual life cycle, and a rhizosphere community consistent with stress-adapted taxa—together reducing the need for large drought-induced transcriptional shifts in leaves. The comparatively small drought DEG set supports a model in which structural and baseline preparedness buffer drought impacts under the conditions tested. Tier 2 (heat) is engaged when temperatures exceed physiological limits and is characterized by a broad transcriptional surge. This response includes strong representation of multiple stress-regulatory transcription factor families (e.g., B3/AP2-ERF and HSF-related factors) and expanded functional/pathway representation linked to metabolism, transport, oxidative stress management, and protein homeostasis. In this framework, facultative CAM may offer an efficient “baseline-plus-surge” strategy—minimizing sustained costs under chronic drought while retaining the capacity for rapid, high-amplitude defense under acute heat episodes.

Overall, our results support *M. forsskalii* as a powerful emerging model for dissecting multi-stress tolerance in facultative CAM species and for identifying genetic elements transferable to crops. The pronounced induction of B3-domain and selected NAC transcription factors under heat, combined with their minimal baseline expression, marks them as high-priority targets for CRISPR-Cas9-mediated enhancement of thermotolerance and water-use efficiency in both C3 and CAM crops. Furthermore, the recently developed and highly reproducible micropropagation protocol for this species provides a practical platform for rapid functional testing of candidate genes, genome editing, and translational breeding programmes aimed at developing climate-resilient varieties for arid and semi-arid agriculture.

## Data Availability

The RNA sequences of the *Mesembryanthemum forskahlii Hochst* shoots in the current study for all samples were submitted to the National Center for Biotechnology Information (NCBI) database under the Sequence Read Archive (SRA) data, with the accession number PRJNA1073520.
